# A Comprehensive Overview of the COVID-19 Literature: Machine Learning–Based Bibliometric Analysis

**DOI:** 10.2196/23703

**Published:** 2021-03-08

**Authors:** Alaa Abd-Alrazaq, Jens Schneider, Borbala Mifsud, Tanvir Alam, Mowafa Househ, Mounir Hamdi, Zubair Shah

**Affiliations:** 1 Division of Information and Computing Technology, College of Science and Engineering Hamad Bin Khalifa University Qatar Foundation Doha Qatar; 2 College of Health and Life Sciences Hamad Bin Khalifa University Qatar Foundation Doha Qatar

**Keywords:** novel coronavirus disease, COVID-19, SARS-CoV-2, 2019-nCoV, bibliometric analysis, literature, machine learning, research, review

## Abstract

**Background:**

Shortly after the emergence of COVID-19, researchers rapidly mobilized to study numerous aspects of the disease such as its evolution, clinical manifestations, effects, treatments, and vaccinations. This led to a rapid increase in the number of COVID-19–related publications. Identifying trends and areas of interest using traditional review methods (eg, scoping and systematic reviews) for such a large domain area is challenging.

**Objective:**

We aimed to conduct an extensive bibliometric analysis to provide a comprehensive overview of the COVID-19 literature.

**Methods:**

We used the COVID-19 Open Research Dataset (CORD-19) that consists of a large number of research articles related to all coronaviruses. We used a machine learning–based method to analyze the most relevant COVID-19–related articles and extracted the most prominent topics. Specifically, we used a clustering algorithm to group published articles based on the similarity of their abstracts to identify research hotspots and current research directions. We have made our software accessible to the community via GitHub.

**Results:**

Of the 196,630 publications retrieved from the database, we included 28,904 in our analysis. The mean number of weekly publications was 990 (SD 789.3). The country that published the highest number of COVID-19–related articles was China (2950/17,270, 17.08%). The highest number of articles were published in bioRxiv. Lei Liu affiliated with the Southern University of Science and Technology in China published the highest number of articles (n=46). Based on titles and abstracts alone, we were able to identify 1515 surveys, 733 systematic reviews, 512 cohort studies, 480 meta-analyses, and 362 randomized control trials. We identified 19 different topics covered among the publications reviewed. The most dominant topic was public health response, followed by clinical care practices during the COVID-19 pandemic, clinical characteristics and risk factors, and epidemic models for its spread.

**Conclusions:**

We provide an overview of the COVID-19 literature and have identified current hotspots and research directions. Our findings can be useful for the research community to help prioritize research needs and recognize leading COVID-19 researchers, institutes, countries, and publishers. Our study shows that an AI-based bibliometric analysis has the potential to rapidly explore a large corpus of academic publications during a public health crisis. We believe that this work can be used to analyze other eHealth-related literature to help clinicians, administrators, and policy makers to obtain a holistic view of the literature and be able to categorize different topics of the existing research for further analyses. It can be further scaled (for instance, in time) to clinical summary documentation. Publishers should avoid noise in the data by developing a way to trace the evolution of individual publications and unique authors.

## Introduction

### Background

In December 2019, Wuhan city in China registered several cases of an unknown disease characterized by pneumonia, dry cough, fatigue, and fever [1]. The investigations revealed that a novel coronavirus (2019-nCoV) was the causative agent of the disease, which was subsequently named COVID-19 [1]. Since then, COVID-19 has spread around the globe, leading the World Health Organization to classify it as a pandemic [2]. This highly contagious pathogen has affected almost every aspect of our daily lives, such as education, traveling, business, transportation, sports, and health care [3]. Most importantly, the COVID-19 pandemic has claimed more than 775,000 lives as of August 19, 2020 [4]. To curb the impact of COVID-19, authorities need to implement effective public health measures related to COVID-19 surveillance, diagnostics, vaccines, treatments, and research [5].

Given the novelty and, consequently, the lack of knowledge about the disease, research can play a crucial role in the fight against the COVID-19 pandemic. Scientists have rapidly mobilized to manage and slowdown the growth of the pandemic. The scientific literature in this domain area has exponentially increased [6,7]. By the end of May 2020, Aristovnik et al [6] and Doanvo et al [8] retrieved 10,344 and 18,412 COVID-19–related publications written in the English language, respectively, from the Scopus database and the COVID-19 Open Research Dataset (CORD-19). In addition, as of July 13, 2020, more than 1711 clinical trials were registered in different clinical trial registries (eg, NCT, EUCTR, and ISRCTN) [9].

It is very important to have a comprehensive overview of the current state of the literature on COVID-19 for several reasons, namely: (1) to organize and coordinate the literature; (2) to explore research topics addressed; (3) to prioritize research needs or gaps; (4) to understand the evolution of the literature; (5) to recognize the leading researchers, institutes, and countries in this area; and (6) to explore connections between research topics and areas.

### Research Problem and Aim

Manually conducting a comprehensive review of the thousands of COVID-19–related publications is a daunting and time-consuming task. Artificial intelligence (AI) methods can play a pivotal role in rapidly surveying the enormous number of publications and extracting critical insights from them. Therefore, in March 2020, the White House strongly recommended researchers to exploit AI methods in COVID-19 research [8].

Several studies have used AI methods to conduct a bibliometric analysis of research on the COVID-19 pandemic [6-8,10-12]. However, we identified the following research gaps in these studies. First, publications analyzed in most studies were dated, approximately to the first three months after the onset of the COVID-19 outbreak; thus, numerous studies published afterward were not analyzed [7,10-16]. Second, several studies analyzed publications related to all types of coronaviruses instead of focusing on COVID-19 [7,10-12,17,18]; hence, the results related to COVID-19 were aggregated with those related to other coronaviruses. Third, several studies included only a few (ranging from 38 to 1482) of the large number of publications related to COVID-19 available in the search period [7,10,12-16,19]. Fourth, most studies did not examine the topics that previous studied had addressed, instead they assessed only the metadata of those studies (eg, countries, authors, number of citations, and published journals) [13,14,16-19]. Fifth, topic identification among various studies was conducted using manual screening instead of AI methods [13-15]. To fill the abovementioned gaps, this study aims to conduct an extensive bibliometric analysis to provide a comprehensive overview of the existing COVID-19 literature.

## Methods

### Study Data Collection

For this study, we used CORD-19, generated by the Allen Institute for AI [20]. The dataset is updated daily to include the latest published articles on COVID-19. We used the update corresponding to the timestamp of July 21, 2020, which contained over 196,630 scholarly articles related to COVID-19 and the coronavirus family of viruses. Allen Institute for AI used the following search terms to retrieve studies on all coronaviruses: “COVID-19” OR “Coronavirus” OR “Corona virus” OR “2019-nCoV” OR “SARS-CoV” OR “MERS-CoV” OR “Severe Acute Respiratory Syndrome” OR “Middle East Respiratory Syndrome”. The search was conducted on PubMed, PubMed Central, and bioRxiv and medRxiv preprint servers. The dataset included a CSV (comma-separate values) file with metadata of all the articles in the dataset, such as article ID, title, abstract, names of authors, and publication date. The articles in the dataset were represented by a single JSON (JavaScript Object Notation) file that consisted of the article ID, title, abstract, body text, and relevant metadata. The metadata of the dataset was analyzed using Python in a Jupyter Notebook environment. We have made our software accessible to the community via GitHub [21]. The CSV metadata file was loaded into a data frame provided by Python’s pandas library. We removed records with empty and non-English abstracts. We also removed duplicate articles and any articles that were published before January 1, 2020. We then used the search terms “novel coronavirus,” “coronavirus 2019,” “2019-nCov,” “COVID-19,” “COVID 2019,” “severe acute respiratory syndrome coronavirus 2,” and “SARS-COV-2” to select only COVID-19–related articles. Thus, we were able to identify a total of 28,904 abstracts of scholarly articles published after January 1, 2020, that were related to COVID-19 for the downstream analysis.

### Data Preprocessing

The 28,904 selected abstracts were cleaned by removing punctuations and alphanumeric characters. Singular and plural uppercased abstract sectioning keywords such as “BACKGROUND,” “OBJECTIVE,” “METHOD,” “RESULT,” and “CONCLUSION” were also removed. The data cleaning was performed using Python programming language in Jupyter Notebook environment. The Python libraries used to clean the data include pandas, NumPy, langdetect, re, string, and TextBlob. The abstracts were then converted to lowercased text. After that, we used the Python Natural Language Toolkit library to tokenize the abstracts and remove the stop words. We then applied the SnowballStemmer model to convert words to their stems. The clean text of the abstracts derived after applying the abovementioned pre-processing steps was used for clustering.

### Document Clustering

For document clustering, we first converted each document (ie, abstract) to a feature vector, where features were defined by term (ie, words) frequency–inverse document frequency (TF-IDF) weights. TF-IDF represents the importance of a word relative to a document in a corpus. This importance increases proportionally to the number of times the word appears in the document but is offset by the frequency of that word in the corpus. This ensures that TF-IDF–based similarity measures between documents are influenced mainly by discriminative words with relatively low frequencies in the corpus [22]. For TF-IDF representation of the abstracts, we used TfidfVectorizer module of the Python scikit library.

The TfidfVectorizer algorithm has two important threshold parameters that cut off low and high word frequencies. The minimum document frequency parameter (min_df) was set to 10 to ignore sporadic terms occurring in less than 10 documents (absolute count). The maximum document frequency parameter (max_df) was set to 0.9 to ignore terms that appear in more than 90% (26,014/28,904) of the documents (relative count). The reason is that we wished to exclude terms that are either too rare to be used in finding document clusters or too common to be discriminative enough to distinguish documents. Based on these parameters (min_df and max_df), the TfidfVectorizer algorithm extracted a vector of 42,061 unique terms to represent each of the 28,904 abstracts, with each term containing a TF-IDF score. This generated a feature matrix of size 28,904×42,061, which was, subsequently, used to feed into a clustering algorithm. We used the k-means clustering algorithm from Python’s scikit library to categorize the abstracts into internally coherent but well-separated clusters. To identify the number of clusters in the k-means clustering algorithm, we used the elbow method to determine the number of clusters in the corpus [23]. Thus, we found 26 to be the optimal number of clusters for this corpus.

## Results

### Search Results

**Figure 1:**
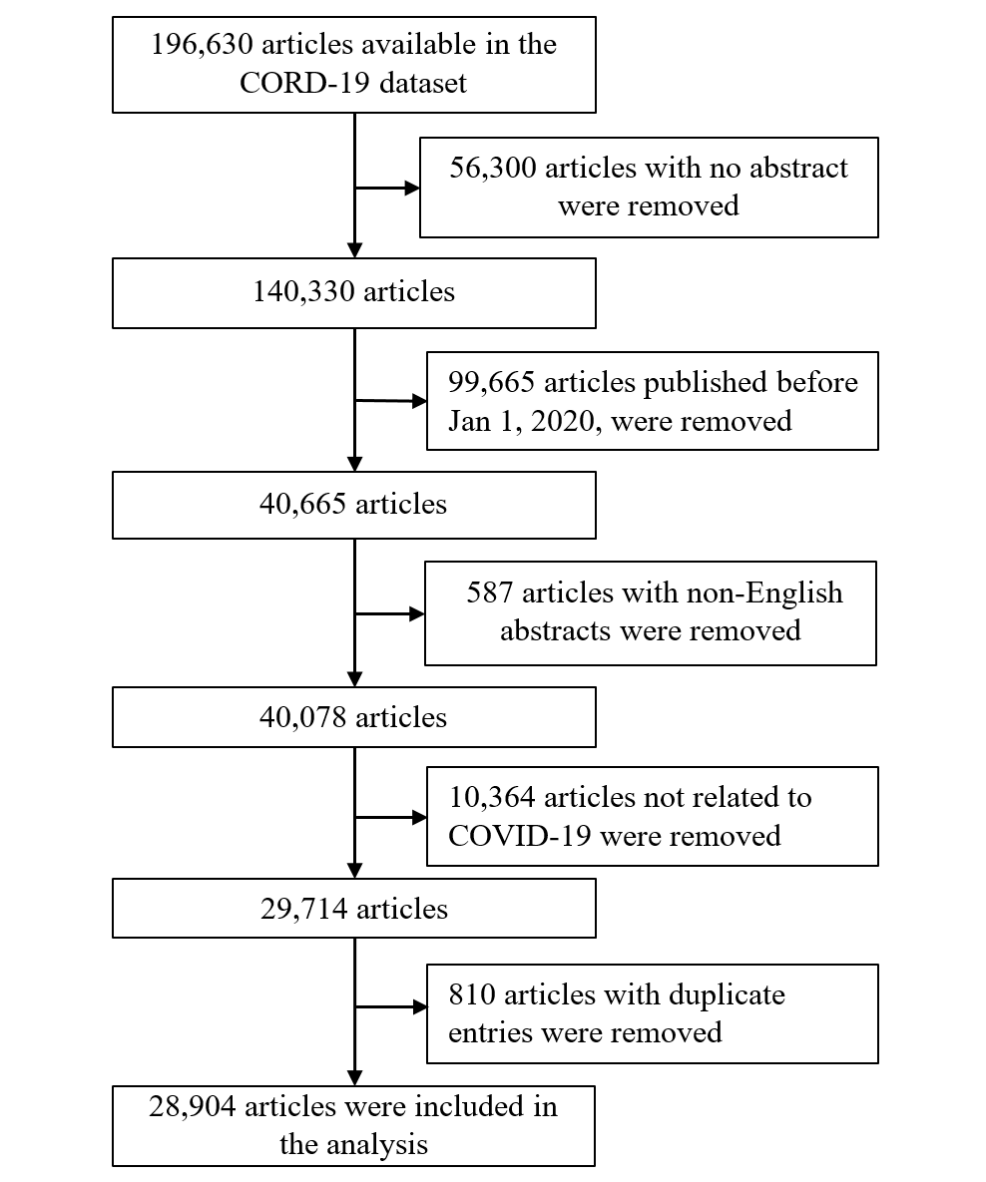
Flowchart of the selection of published articles. CORD-19: COVID-19 Open Research Dataset.

By July 21, 2020, the CORD-19 dataset comprised 196,630 articles (Figure 1). Of those, we excluded 167,726 articles for the following reasons: (1) abstracts were unavailable (n=56,300); (2) the articles were published before January 1, 2020 (n=99,665); (3) the articles were written in a language other than English (n=587); (4) the articles were not related to COVID-19, as their titles and abstracts did not contain our search terms (n=10,364); and (5) they had duplicate entries (n=810). Consequently, we included 28,904 articles in the analysis in this study.

### Characteristics of Publications

**Figure 2:**
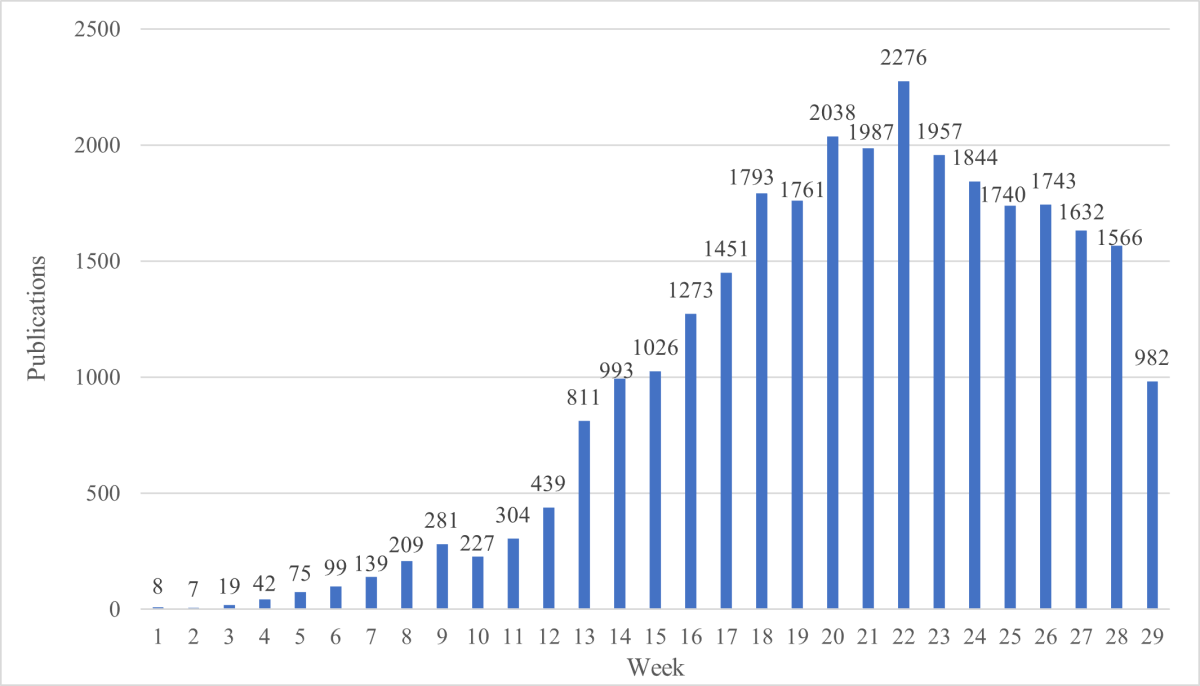
Number of publications in each week (2020).

The first paper was published on January 2, 2020. As shown in Figure 2, the number of publications in each week increased considerably since then, until a peak was reached in week 22 (2276 publications). Thereafter, the number of research papers published began to decrease. The mean number of publications for each week was 990 (SD 789.3). The country of publication was identified for 17,270 publications, which were conducted across 221 countries and territories. The country that published the highest number of articles was China (2950/17,270, 17.08%), followed by the United States (1357/17,270, 7.86%), Italy (1157/17,270, 6.70%), Saudi Arabia (978/17,270, 5.66%), and India (854/17,270, 4.94%) (Table 1).

The selected articles were published in about 2500 journals. The highest number of articles were published in bioRxiv (n=1374), the most prominent preprint server for biology. The top 10 sites for publishing COVID-19–related articles (journals and preprint servers) are shown in Table 2. The publications included in this analysis were authored by 150,600 authors. Among those authors, Lei Liu published the highest number of articles (n=46; see Table 3). Based on titles and abstracts alone, we were able to identify 1515 surveys, 733 systematic reviews, 512 cohort studies, 480 meta-analyses, 362 randomized control trials, 199 case studies, 79 scoping reviews, and 62 case-control studies (Table 4). Note that these numbers include only the top 8 study methods for those publications that mention the study method in either the abstract or the title.

**Table 1:** Top 10 countries (N=221) by the number of COVID-19–related publications (N=17,270).

Rank	Country	Publications, n (%)
1	China	2950 (17.08)
2	United States	1357 (7.86)
3	Italy	1157 (6.70)
4	Saudi Arabia	978 (5.66)
5	India	854 (4.94)
6	Canada	671 (3.89)
7	United Kingdom	525 (3.04)
8	Germany	449 (2.60)
9	Australia	403 (2.33)
10	France	383 (2.22)

**Table 2:** Top 10 sites (journals and preprint servers) where COVID-19–related articles (N=28,904) were published.

Rank	Journal or server	Publications, n (%)
1	bioRxiv	1374 (4.75)
2	Journal of Medical Virology	468 (1.6)
3	medRxiv	340 (1.18)
4	International Journal of Environmental Research and Public Health	223 (0.77)
5	Clinical Infectious Diseases	195 (0.67)
6	International Journal of Infectious Diseases	184 (0.64)
7	Science of the Total Environment	165 (0.57)
8	Cureus	148 (0.51)
9	Psychological Trauma: Theory, Research, Practice, and Policy	138 (0.48)
10	Medical Hypotheses	136 (0.47)

**Table 3:** Top 10 authors (N=15,600) by the number of COVID-19–related publications.

Rank	Author name	Publications, n	Author affiliation
1	Lei Liu	46	Southern University of Science and Technology, China
2	Kwok-Yung Yuen	34	The University of Hong Kong, China
3	Christian Drosten	31	Charité University Medicine Berlin, Germany
4	Ralph S Baric	31	University of North Carolina at Chapel Hill, USA
5	Gerardo Chowell	30	Georgia State University, USA
6	Hongzhou Lu	30	Fudan University, Shanghai, China
7	Giuseppe Lippi	29	University of Verona, Italy
8	Jasper Fuk-Wo Chan	27	The University of Hong Kong, China
9	Kelvin Kai-Wang To	25	The University of Hong Kong, China
10	Valerie A Canady	23	Mental Health Weekly, USA

**Table 4:** Eight most common study methods extracted from the publications mentioning the study design used in the abstract.

Rank	Study method	Publications, n
1	Survey	1515
2	Systematic review	733
3	Cohort study	512
4	Meta-analysis	480
5	Randomized control trial	362
6	Case study	199
7	Scoping review	79
8	Case-control study	62

### Results of Topics Modelling

#### Overview

The analysis generated 26 clusters from the included publications. We were able to identify the topic of 21 clusters, whereas the remaining 5 clusters were not labeled as they contained publications with very diverse topics that belonged to other clusters. Therefore, publications in these 5 clusters were moved to the most appropriate cluster among the 21 clusters. Four of the 21 clusters contained publications addressing only two different topics; thus, we further merged the 4 clusters to form 2 different clusters. Overall, we identified 19 different topics addressed in the included publications (Table 5).

**Table 5:** COVID-19–related topics addressed by the included publications (N=28,904).

Number	Topic	Articles, n (%)	Top 20 unigrams
1	Public health response	5393 (18.66)	covid, pandemic, health, coronavirus, disease, public, data, world, spread, social, study, global, outbreak, countries, cases, measures, virus, sars, results, people
2	Clinical care practices for patients during the COVID-19 pandemic	5118 (17.71)	covid, pandemic, patients, care, disease, coronavirus, health, patient, infection, risk, healthcare, clinical, sars, cov, management, medical, respiratory, hospital, cases, acute
3	Clinical characteristics and risk factors of COVID-19	3313 (11.46)	covid, patients, disease, coronavirus, severe, clinical, study, results, age, data, risk, respiratory, hospital, sars, infection, cov, cases, acute, mortality, higher
4	Epidemic models for COVID-19 spread	2964 (10.25)	covid, model, data, cases, number, epidemic, disease, time, results, spread, pandemic, coronavirus, based, countries, infected, infection, rate, study, measures, outbreak
5	Therapies and vaccines for COVID-19	1845 (6.38)	sars, cov, covid, drug, coronavirus, drugs, viral, antiviral, virus, potential, disease, pandemic, respiratory, treatment, protease, molecular, compounds, severe, based, inhibitors
6	Host immune response	1837 (6.36)	covid, cov, sars, disease, coronavirus, infection, severe, respiratory, patients, acute, syndrome, immune, viral, cells, virus, clinical, inflammatory, response, pandemic, cell, cytokine
7	Diagnosis of COVID-19 using PCR^a^	1602 (5.54)	cov, sars, covid, pcr, positive, coronavirus, testing, respiratory, results, patients, rt, disease, infection, detection, samples, test, clinical, viral, time, negative
8	Mental health and disorders during the COVID-19 pandemic	915 (3.17)	covid, health, mental, pandemic, anxiety, psychological, study, coronavirus, stress, depression, results, social, disease, risk, impact, related, people, survey, symptoms, outbreak
9	Diagnosis of COVID-19 based on chest imaging	874 (3.02)	covid, ct, patients, chest, disease, coronavirus, pneumonia, clinical, diagnosis, results, imaging, findings, lung, cases, ground, glass, tomography, computed, features, images
10	Social distancing measures	868 (3)	covid, social, distancing, pandemic, measures, spread, contact, disease, data, health, model, number, transmission, cases, control, population, coronavirus, time, tracing, public
11	Virus genomics	816 (2.82)	sars, cov, coronavirus, virus, genome, covid, viral, analysis, sequences, respiratory, severe, pandemic, human, disease, sequence, acute, syndrome, genomes, china, study
12	Protein structures of 2019-nCoV^b^	706 (2.44)	cov, sars, spike, protein, binding, coronavirus, covid, receptor, virus, human, viral, pandemic, ace, domain, cell, vaccine, infection, disease, host, development
13	Host cell entry	584 (2.02)	ace, sars, cov, covid, angiotensin, enzyme, converting, receptor, coronavirus, disease, infection, expression, respiratory, cells, severe, human, syndrome, entry, cell, virus
14	Clinical care practices for patients with cancer	441 (1.53)	cancer, covid, patients, pandemic, treatment, risk, care, disease, coronavirus, infection, health, clinical, patient, management, cov, sars, severe, therapy, high, oncology
15	Detection of 2019-nCoV antibodies	411 (1.42)	cov, sars, igg, covid, antibodies, patients, antibody, igm, infection, results, positive, disease, coronavirus, samples, study, test, assay, serological, sensitivity, clinical
16	Personal protective equipment	350 (1.21)	covid, masks, mask, pandemic, protective, equipment, face, personal, respirators, health, coronavirus, healthcare, workers, sars, cov, respiratory, surgical, disease, study, ppe
17	Diabetes mellitus and COVID-19	336 (1.16)	covid, pandemic, patients, diabetes, disease, care, risk, coronavirus, health, nursing, nurses, infection, management, severe, results, data, study, clinical, high, methods
18	Pregnancy and childbirth during the COVID-19 pandemic	312 (1.08)	women, pregnant, covid, pregnancy, infection, sars, coronavirus, cov, disease, severe, clinical, maternal, patients, cases, data, transmission, respiratory, pandemic, delivery, results
19	Organ transplantation during the COVID-19 pandemic	219 (0.76)	covid, transplant, recipients, patients, disease, coronavirus, pandemic, cov, sars, infection, transplantation, kidney, severe, organ, clinical, risk, respiratory, acute, liver, patient

^a^PCR: polymerase chain reaction.

^b^2019-nCoV: novel coronavirus.

#### Topic 1: Public Health Response

This topic was addressed by 18.66% (5393/28,904) of the publications. The publications in this cluster mainly discussed how public health authorities in various countries responded to the COVID-19 pandemic (eg, [24-28]). The top 5 authors in terms of the highest number of publications related to this topic were Claudine McCarthy (n=8), Valerie A Canady (n=6), Alison Knopf (n=6), Alimuddin Zumla (n=6), and Nima Rezaei (n=6). The top 5 journals and preprint servers hosting the highest number of publishing articles related to this topic were the International Journal of Environmental Research and Public Health (n=82), Science of the Total Environment (n=80), New Scientist (n=56), Journal of Medical Virology (n=53), and bioRxiv (n=50). The first paper related to this topic was published on January 10, 2020. The number of publications in each week increased significantly until it reached a peak in week 23 (n=434); it then decreased noticeably (Multimedia Appendix 1). The mean number of weekly publications in this cluster was 183.6 (SD 151.5).

#### Topic 2: Clinical Care Practices During the COVID-19 Pandemic

A total of 17.71% (5118/28,904) of all included publications were mainly about clinical care practices for non–COVID-19 patients during the COVID-19 pandemic (eg, [29-33]). The following authors published the highest number of publications related to this topic: Karthik Rajasekaran (n=14), Francesco Esperto (n=12), Raju Vaishya (n=9), Namrata Sharma (n=8), and Santosh G Honavar (n=8). The top 5 journals publishing articles related to this topic were Otolaryngology-Head and Neck Surgery (n=115), the Journal of the European Academy of Dermatology and Venereology (n=45), Cureus Journal of Medical Science (n=41), Anaesthesia (n=40), and World Neurosurgery (n=35). In this cluster, the first article was published on January 3, 2020. There was a considerable rise in the number of weekly publications from week 12 until it reached a peak in week 23 (n=479); this was followed by a sharp decrease (Multimedia Appendix 1). The mean number of weekly publications in this cluster was 175.2 (SD 159.6).

#### Topic 3: Clinical Characteristics and Risk Factors of COVID-19

This topic was discussed in 11.46% (3313/28,904) of the publications (eg, [34-38]). The top 5 authors who published the highest number of publications in this cluster were Lei Liu (n=25), Lanjuan Li (n=13), Jifang Sheng (n=10), Giuseppe Lippi (n=9), and Nanshan Zhong (n=9). The top 5 journals and preprint servers publishing articles related to this topic were the Journal of Medical Virology (n=109), medRxiv (n=57), Cureus Journal of Medical Science (n=35), Clinical Infectious Diseases (n=33), and the International Journal of Infectious Diseases (n=32). The first article related to this topic was published on January 17, 2020. The mean number of weekly publications in this cluster was 113.7 (SD 98.9), and the highest number of weekly publications was 266 in week 20 (Multimedia Appendix 1).

#### Topic 4: Epidemic Models for COVID-19 Spread

A total of 10.25% (2964/28,904) of the included publications were related to this topic (eg, [39-43]). The 5 most prominent authors in this cluster were Gerardo Chowell (n=22), Benjamin J Cowling (n=18), Kenji Mizumoto (n=14), Shi Zhao (n=13), and Rosalind M Eggo (n=13). The most common journals and preprint servers where the articles related to this topic were published included Chaos, Solitons & Fractals (n=73), medRxiv (n=66), the International Journal of Infectious Diseases (n=36), Zhonghua liuxingbingxue zazhi (n=30), and bioRxiv (n=26). The first paper related to this topic was published on the January 19, 2020. Although there was a sharp increase in the number of weekly publications between weeks 12 and 15, the trend was almost stable from week 15 to week 22. Then, a rapid decline in the number of weekly publications was noticed (Multimedia Appendix 1). The mean number of weekly publications in this cluster was 101.6 (SD 68.2).

#### Topic 5: Therapies and Vaccines for COVID-19

In all, 6.38% (1845/28,904) of the publications were about the development and repurposing of therapies and vaccines for COVID-19 (eg, [44-47]). The following authors published the highest number of articles related to this topic: Wei Zhang (n=13), Xiuna Yang (n=9), Haitao Yang (n=9), Zihe Rao (n=9), and Yao Zhao (n=8). The journals and preprint servers publishing the highest number of studies in this cluster were bioRxiv (n=174), the Journal of Biomolecular Structure and Dynamics (n=74), Trials (n=49), the Journal of Medical Virology (n=20), and Clinical Pharmacology & Therapeutics (n=14). In this cluster, the first article was published on January 6, 2020. The number of weekly publications increased dramatically from week 14 until a peak was reached in week 22 (n=144); thereafter, it decreased noticeably (Multimedia Appendix 1). The mean number of weekly publications in this cluster was 62.9 (SD 52.3).

#### Topic 6: Host Immune Response to 19-nCoV

This topic was discussed in about 6.36% (1837/28,904) of the publications (eg, [48-52]). Authors who had the highest number of publications related to this topic were Alessandro Sette (n=7), Stanley Perlman (n=6), Nima Rezaei (n=6), Irfan Rahman (n=5), and Akiko Iwasaki (n=6). The top 5 journals and preprint servers in terms of publishing articles related to this topic were bioRxiv (n=199), Medical Hypotheses (n=50), the Journal of Medical Virology (n=49), Frontiers in Immunology (n=22), and the British Journal of Haematology (n=19). The earliest article related to this topic was published on January 2, 2020. From that date until week 14, there was a slight increase in the number of weekly publications before it increased markedly, peaking in week 25 (n=155) (Multimedia Appendix 1). The mean number of weekly publications in this cluster was 62.8 (SD 54.6).

#### Topic 7: Diagnosis of COVID-19 Using Polymerase Chain Reaction

Using polymerase chain reaction (PCR) for diagnosing COVID-19 was a key topic discussed in 5.54% (1602/28,904) of the publications (eg, [53-57]). The most common authors writing about this topic were Alexander L Greninger (n=11), Kwok-Yung Yuen (n=10), Jasper Fuk-Woo Chan (n=9), Kelvin Kai-Wang To (n=9), and Cyril Chik-Yan Yip (n=9). The top 5 journals and preprint servers that published the highest number of studies related to this topic were bioRxiv (n=136), the Journal of Medical Virology (n=53), the Journal of Clinical Virology (n=40), medRxiv (n=31), and Clinical Infectious Diseases (n=30). The earliest study in this cluster was published at the beginning of week 3. The highest number of weekly publications was 119 in weeks 22 and 23 (Multimedia Appendix 1). The mean number of weekly publications related to this topic was 54.8 (SD 45.6).

#### Topic 8: Mental Health and Disorders During the COVID-19 Pandemic

This topic is about COVID-19–related mental health and disorders, which was explored by 3.17% (915/28,904) of the publications (eg, [58-62]). The top 5 authors in terms of number of publications related to this topic were Valerie A Canady (n=15), Mark D Griffiths (n=8), Stephen X Zhang (n=6), Zhilei Shang (n=5), and Modesto Leite Rolim Neto (n=5). The top 5 journals publishing studies related to this topic were Psychological Trauma: Theory, Research, Practice, and Policy (n=101); Psychiatry Research (n=48); the International Journal of Environmental Research and Public Health (n=37); the Journal of Affective Disorders (n=23); and Mental Health Weekly (n=23). In this cluster, the first article was published at the beginning of week 8. There was a considerable rise in the number of weekly publications from week 14 until a peak was reached in week 23 (n=94); this was followed by a steep decline (Multimedia Appendix 1). The mean number of weekly publications in this cluster was 31.1 (SD 30.9).

#### Topic 9: Diagnosis of COVID-19 Based on Chest Imaging

Diagnosis of COVID-19 based on chest imaging (eg, x-ray and computed tomography) was the main topic in 3.02% (874/28,904) of the included publications (eg, [63-67]). The 5 most prominent authors in this topic were Liming Xia (n=14), Michael Chung (n=12), Hongjun Li (n=11), Dinggang Shen (n=11), and Fuhua Yan (n=11). The top 5 journals in this cluster were European Radiology (n=38), Radiology (n=21), the American Journal of Roentgenology (n=17), Radiology: Cardiothoracic Imaging (n=16), and Clinical Nuclear Medicine (n=16). The first article about this topic was published on February 3, 2020. The mean number of weekly publications in this cluster was 29.9 (SD 21.4), and the highest number of weekly publications was 60 in week 18 (Multimedia Appendix 1).

#### Topic 10: Social Distancing Measures

A total of 3% (868/28,904) of the articles discussed the topic of social distancing measures used to fight against the COVID-19 pandemic (eg, [68-72]). Authors who had the highest number of publications related to this topic were Lei Zhang (n=7), Adam J Kucharski (n=6), Amy Gimma (n=5), Gerardo Chowell (n=5), and Petra Klepac (4). The top 5 journals and preprint servers in terms of publishing articles related to this topic were medRxiv (n=28); Chaos, Solitons & Fractals (n=6); Morbidity and Mortality Weekly Report (n=5); Science (n=5); and Disaster Medicine and Public Health Preparedness (n=5). The earliest article related to this topic was published in week 7. There was a dramatic rise in the number of weekly publications between week 12 and week 19; thereafter, the trend was unstable from week 20 to week 29 (Multimedia Appendix 1). The mean number of weekly publications in this cluster was 29.8 (SD 26.8).

#### Topic 11: Virus Genomics

Around 2.82% (816/28,904) of the publications were about genome sequences of 2019-nCoV (eg, [73-77]). The most common authors writing about this topic were Massimo Ciccozzi (n=11), Andrew Rambaut (n=11), Marta Giovanetti (n=10), Silvia Angeletti (n=10), and Domenico Benvenuto (n=9). The top 5 journals and preprint servers that published the highest number of studies in this cluster were bioRxiv (n=295); the Journal of Medical Virology (n=32); Microbiology Resource Announcements (n=14); Viruses (n=13); and Infection, Genetics and Evolution (n=12). The earliest study in this cluster was published at the beginning of week 4. The mean number of weekly publications in this cluster was 27.7 (SD 18.2). The highest number of weekly publications was 56 in week 24 (Multimedia Appendix 1).

#### Topic 12: Protein Structures of 2019-nCoV

About 2.44% (706/28,904) of the included publications focused on structures and functions of 2019-nCoV proteins (eg, [78-82]). The top 5 authors in terms of the number of publications related to this topic were Ralph S Baric (n=14), Jason S McLellan (n=12), Shibo Jiang (n=10), James Brett Case (n=10), and Daniel Wrapp (n=10). The journals and preprint servers publishing the highest number of studies in this cluster were bioRxiv (n=333), the Journal of Virology (n=15), the Journal of Biomolecular Structure & Dynamics (n=15), Science (n=13), and the Journal of Medical Virology (n=11). The earliest study related to this topic was published on January 3, 2020. The mean number of weekly publications in this cluster was 24.2 (SD 18.6). The highest number of weekly publications was 68 in week 25 (Multimedia Appendix 1).

#### Topic 13: Host Cell Entry

Host cell entry for 19-nCoV (via angiotensin-converting enzyme 2) was a key topic discussed in 2.02% (584/28,904) of the reviewed publications (eg, [83-87]). The 5 most prominent authors in this cluster were Serpil Erzurum (n=4), Giuseppe Lippi (n=4), Daniel Batlle (n=4), Hong Gao (n=4), and Claudio Cavallini (n=3). The most common journals and preprint servers in this cluster were bioRxiv (n=117), the Journal of Medical Virology (n=11), Medical Hypotheses (n=10), European Respiratory Journal (n=8), and medRxiv (n=7). The first article related to this topic was published in the mid of week 4. The number of weekly publications was almost stable between weeks 4 and 13. Thereafter, a sharp increase was noticed between weeks 14 and 16, but it was not stable from then until week 29 (Multimedia Appendix 1). The number of publications in week 20 was the highest (n=50). The mean number of weekly publications in this cluster was 19.9 (SD 16.6).

#### Topic 14: Patients With Cancer During the COVID-19 Pandemic

A total of 1.53% (441/28,904) of the included publications were about patients with cancer during the COVID-19 pandemic (eg, [88-91]). The following authors published the highest number of articles related to this topic: Solange Peters (n=6), Umberto Ricardi (n=5), Conghua Xie (n=5), Giuseppe Curigliano (n=5), and Alessio Cortellini (n=4). The top 5 journals in terms of publishing articles related to this topic were Head & Neck (n=16), ecancermedicalscience (n=15), Radiotherapy and Oncology (n=10), Advances in Radiation Oncology (n=8), and Cancer Discovery (n=8). From week 1 to 7, only 1 study related to this topic was published. The highest number of weekly publications was in week 22 (n=49), but since then, the number of weekly publications declined steeply (Multimedia Appendix 1). The mean number of weekly publications related to this topic was 15.2 (SD 14.4).

#### Topic 15: Detection of 2019-nCoV Antibodies

Detection of antibodies against 2019-nCoV using serological assays was a topic discussed in 1.42% (411/28,904) of all publications (eg, [92-96]). The top 5 authors writing about this topic were Florian Krammer (n=6), Jing Wang (n=6), Yong Zhang (n=5), Juan Chen (n=5), and Viviana Simon (n=5). The top 5 journals and preprint servers that published the highest number of studies in this cluster were bioRxiv (n=30), medRxiv (n=20), the Journal of Medical Virology (n=18), the Journal of Clinical Virology (n=14), and the Journal of Clinical Microbiology (n=7). Only 1 study in this cluster was published in the first 6 weeks. There was a dramatic increase in the number of weekly publications between weeks 16 and 21. Although the number of weekly publications slightly decreased from week 22 until week 26, it increased rapidly until reaching the peak in weeks 28 and 29 (n=36) (Multimedia Appendix 1). The mean number of weekly publications in this cluster was 14.1 (SD 12.8).

#### Topic 16: Personal Protective Equipment

Around 1.21% (350/28,904) of the publications focused on personal protective equipment in the COVID-19 era (eg, [97-100]). Authors who published the most in this cluster were Holly Seale (n=3), Keith K Wannomae (n=3), Lei Liao (n=3), Wang Xiao (n=3), and Steven Chu (n=3). The highest numbers of studies were published in the following journals and preprint servers: the American Journal of Infection Control (n=8), the Journal of Hospital Infection (n=7), Anaesthesia (n=6), the Journal of the European Academy of Dermatology and Venereology (n=6), ACS Nano (n=5), and medRxiv (n=5). No articles in this cluster were published before week 9. The mean number of weekly publications in this cluster was 11.9 (SD 11.6). The highest number of weekly publications was 34 in week 23 (Multimedia Appendix 1).

#### Topic 17: Diabetes Mellitus and COVID-19

Health care management, clinical characteristics, and risk factors for mortality of COVID-19 patients with diabetes was discussed in 1.16% (336/28,904) of the included articles (eg, [101-104]. The 5 most prominent authors in this cluster were Hui Wang (n=5), Sam Foster (n=4), Anoop Misra (n=4), Béatrice Bouhanick (n=3), and Kamlesh Khunti (n=3). The most common journals in this cluster were Diabetes Research and Clinical Practice (n=20), Diabetology & Metabolic Syndrome (n=17), the British Journal of Nursing (n=10), the Journal of the American Medical Directors Association (n=7), and Diabetes Technology & Therapeutics (n=6). Only 4 articles related to this topic were published between weeks 1 and 12. However, there was a substantial increase in the number of weekly publications from week 17 until the peak was reached in week 20 (n=35); this was followed by a slight decrease (Multimedia Appendix 1). The mean number of weekly publications in this cluster was 11.4 (SD 11.6).

#### Topic 18: Pregnancy and Childbirth During the COVID-19 Pandemic

About 1.08% (312/28,904) of the publications focused on numerous aspects of pregnancy and childbirth during the COVID-19 pandemic (eg, [105-109]). The most common authors writing about this topic were Ling Feng (n=7), Jiafu Li (n=6), Olivier Picone (n=5), Dunjin Chen (n=5), and Guoqiang Sun (n=5). The top 5 journals in terms of publishing articles in this cluster were the International Journal of Gynaecology and Obstetrics (n=18), The Journal of Maternal-Fetal & Neonatal Medicine (n=16), the American Journal of Obstetrics and Gynecology (n=20), Obstetrics and Gynecology (n=10), and the American Journal of Perinatology (n=9). The earliest article in this cluster was published on February 10, 2020. The mean number of weekly publications related to this topic was 10.8 (SD 8.9), and the highest number of weekly publications was 27 in week 21 (Multimedia Appendix 1).

#### Topic 19: Organ Transplantation During the COVID-19 Pandemic

Organ transplantation in the era of COVID-19 was a key topic in 0.76% (219/28,904) of the included articles (eg, [110-113]) The top 5 authors in terms of number of publications related to this topic were Paolo Cravedi (n=4), Zhishui Chen (n=4), Luciano De Carlis (n=4), Lai Wei (n=4), and Ashley Fan (n=3). The top 5 journals in terms of publishing articles related to this topic were the American Journal of Transplantation (n=69), Transplant Infectious Disease (n=35), Transplant International (n=11), Transplantation Proceedings (n=10), and Liver Transplantation (n=5). Only one study in this cluster was published before week 12. The mean number of weekly publications related to this topic was 7.5 (SD 8.3), and the highest number of weekly publications was 26 in week 24 (Multimedia Appendix 1).

## Discussion

### Principal Findings

We found that 5.92% (1714/28,904) of the included published articles were hosted on preprint servers (bioRxiv or medRxiv). Although these servers are not the only preprint servers available in the academic publishing landscape (many journals publish articles online before they go into print, and we have also observed a rise of purely online journals), they are indicative of the pace with which new knowledge is made available by the international research community. Since such preprint servers do not undergo formal peer reviewing and are, thus, not regarded publications in the traditional academic sense, many researchers are using this device to make findings available and to solicit feedback from the international community before undergoing formal peer-reviewing by journals—a process that takes at least 2 months to get the submitted paper published.

Among the peer-reviewed journals, the Journal of Medical Virology has published the highest number of COVID-19–related articles (n=468). Aristovnik et al and Hossain also listed the Journal of Medical Virology in the top-5 journals publishing COVID-19–related articles [6,14]. The Journal of Medical Virology clearly stands out, as it has published more than twice the number of papers compared to the second-ranked journal—the International Journal of Environmental Research and Public Health (n=223). Aristovnik et al [6] listed the International Journal of Environmental Research and Public Health among the 10 top-ranked journals based on COVID-19–related research articles [6]. The source normalized impact per paper (SNIP), in the year 2019, was 0.780 for the Journal of Medical Virology [114] and 1.248 for the International Journal of Environmental Research and Public Health [115], and the average time from the submission to the first decision was about 6 weeks [116] and 3 weeks [117], respectively. We believe the speed of the reviewing process of these journals may have motivated the authors to submit their work to these journals.

Considering the study methods, we found that the highest number of studies (n=1515) were surveys, followed by reviews (systematic review, scoping review, or meta-analyses), as shown in Table 3. As the number of research studies on COVID-19 is rapidly increasing, review articles are of utmost importance to summarize the ongoing effort and progress to combat against COVID-19. We found case-control studies to be the lowest represented study design (n=62 only). We speculate that the lack of available data was the main reason for the scarcity of this type of research study. Interestingly, 362 randomized control trials in 7 months indicate the enormous effort made by the scientific community to combat this pandemic.

Furthermore, we grouped the 19 topics addressed in the included studies into six thematic areas (summarized in Table 6). The dominant thematic clusters were “Clinical aspects” (29.17%) and “Epidemiology” (28.91%). The “Clinical aspects” theme covers multiple aspects of the clinical practices for patient care and risk factors related to COVID-19. It consists of two topics (ie, “clinical care practices for patients during the COVID-19 pandemic” and “clinical characteristics and risk factors of COVID-19). Interestingly, the “Epidemiology” theme also comprises only two topics (ie, “Public health response” and “Epidemic models for COVID-19 spread”), further underscoring the dominance of these topics.

**Table 6:** Topics grouped by thematic cluster, including the percentage of articles by topic and cluster.

Thematic cluster, topic number, and title				Articles (N=28,904), n		Topic dominance (%)		Cluster dominance (%)
**Clinical aspects**								29.17
	(2)	Clinical care practices for patients during the COVID-19 pandemic	5118		17.71			
	(3)	Clinical characteristics and risk factors of COVID-19	3313		11.46			
**Epidemiology**								28.91
	(1)	Public health response	5393		18.66			
	(4)	Epidemic models for COVID-19 spread	2964		10.25			
**Therapeutics**								21.03
	(5)	Therapies and vaccines for COVID-19	1845		6.38			
	(6)	Host immune response	1837		6.36			
	(11)	Virus genomics	816		2.82			
	(12)	Protein structures of 2019-nCoV	706		2.44			
	(13)	Host cell entry	584		2.02			
**Diagnostics**								9.98
	(7)	Diagnosis of COVID-19 using PCR	1602		5.54			
	(9)	Diagnosis of COVID-19 based on chest imaging	874		3.02			
	(15)	Detection of 2019-nCoV antibodies	411		1.42			
**Related conditions**								7.70
	(8)	Mental health and disorders during the COVID-19 pandemic	915		3.17			
	(14)	Patients with cancer during the COVID-19 pandemic	441		1.53			
	(17)	Diabetes mellitus and COVID-19	336		1.16			
	(18)	Pregnancy and childbirth during the COVID-19 pandemic	312		1.08			
	(19)	Organ transplantation during the COVID-19 pandemic	219		0.76			
**Prevention**								4.21
	(10)	Social distancing measures	868		3			
	(16)	Personal protective equipment	350		1.21			

The third most prominent theme “Therapeutics” (21.03%) comprises five topics, making it the most diverse theme; the topics in this theme range from “host cell entry” to drug discovery–related terms such as “Protein structures of 2019-nCoV” and “Virus genomics,” as well as “Therapies and vaccines for COVID-19.” This theme highlights the initiatives of the scientific community to discover drugs and vaccines and understand the underlying virus-host mechanism to pave the way for effective therapeutic solutions for COVID-19. Considering the severity of COVID-19, we believe there still may be a lack of publications in this theme, despite comprising slightly more than 20% of all articles. We believe that, as clinical practices and public health responses mature, this theme will receive more research articles in the near future.

Almost 10% of articles form the “Diagnostics” theme. This theme focuses on the diagnosis of COVID-19 based on PCR, radiological images, or antibodies. PCR is among the most accurate technologies to diagnose COVID-19 [114], which explains the numerous relevant publications. Due to the advancement of deep learning techniques, radiological image-based diagnosis is becoming more effective and has the potential to save time in clinical environments [115]. As a result, we observed a large number of publications on radiological image-based analyses, which is captured under the topic “Diagnosis of COVID-19 based on chest imaging.” Antibodies, developed in hosts combatting the novel coronavirus, can be considered a detection mechanism that may play an important role complementary to PCR testing [116]. It can be very effective for the diagnosis of patients with asymptomatic COVID-19 or negative RT–PCR results [117]. We also noticed many publications on antibody responses against COVID-19, which are covered by the topic “Detection of 2019-nCoV antibodies”.

The interplay between COVID-19 and related medical conditions is captured by the “Related conditions” theme. This theme not only comprises articles discussing related conditions caused by COVID-19 but also other conditions that may elevate the COVID-19 risk for the patients with those conditions. About 8% of all articles fall into this theme, covering topics such as mental disorder, diabetes, cancer, pregnancy, childbirth complications, and organ transplantation.

Only slightly more than 4% of all articles fall into the “Prevention” theme. This may be surprising, since prevention is of utmost importance while vaccines and treatments are still under development. However, we believe that this theme is not covered by more studies due to the recent wide acceptance and effectiveness of social distancing and personal protective equipment. Consequently, we expect the percentage of articles grouped in this theme to further reduce in the future. Further insights into the research landscape and the shift in themes over time is summarized in Multimedia Appendix 2.

We noticed that biomedical informatics had a crucial role in several topics. For instance, clinical decision support systems were used in many studies to diagnose COVID-19 based on chest imaging. Telemedicine was also used in multiple studies to provide the required health care support for the patients during the COVID-19 pandemic. Further, mobile applications, including contact tracing apps, were one of the main social distancing measures described in these studies. AI-based models were used in multiple studies to predict protein structures of 2019-nCoV to understand the underlying mechanism of drug-target interaction. Many studies proposed novel AI-based models to discover COVID-19 drugs and vaccines and repurpose existing drugs approved by the Food and Drug Administration as a part of the treatment plan for COVID-19.

### Strengths and Limitations

#### Strengths

To the best of our knowledge, our study covers the largest collection of COVID-19–related articles (N=28,904, after considering the inclusion and exclusion criteria) published in the period of 7 months (January to mid-July 2020). The main strength of this study is that it demonstrates the feasibility of mostly automated, AI-based data mining at scale. We believe this is of utmost importance because articles on COVID-19 are published faster than nonautomatic surveys can organize, analyze, and present them to the scientific community.

#### Limitations

All the publications were collected from only one specific database (CORD-19), so we may have missed some studies or preprints that were not considered in this database. Given the substantial number of publications included in our analysis, we are confident that a large part of the COVID-19 literature was covered. Further, we did not conduct a detailed manual analysis of studies published in journals such as Journal of Medical Internet Research and the International Journal of Medical Informatics to evaluate the use of eHealth technologies for COVID-19, which we believe is beyond the scope of our work and requires other study methodologies such as systematic or scoping review. However, readers are referred to several studies and reviews, which have been conducted to explore eHealth technologies used in the fight against the COVID-19 pandemic [118-121].

We only considered the articles published in the English language, which may introduce some bias in our analysis. Additionally, articles published after mid-July 2020 were not considered in this study. Moreover, due to the inherent limitation of the bibliographic analysis, which enabled high-level profiling of the text from the corpus of literature, we cannot provide any evidence-based solution for the diagnosis and treatment of COVID-19. Further analysis of the articles that fall under each topic should be considered more carefully.

For this study, we only analyzed article titles, abstracts, and author data. As a result, we could not identify the country of publication for 11,634 articles (around 40%). Although we removed duplicates, academic publications undergo subtle morphology changes. These come in the form of revisions, preprints, follow-up studies, among others. Given only the abstract and title were screened, we cannot rule out that some publications may have substantial overlaps. Finally, there exist ambiguities with respect to author and journal names because authors with the same name can only be resolved uniquely by affiliation or, in some cases, other identifiers such as ORCID. If such elucidating identifiers are missing, automatic disambiguation is not possible. Likewise, journals may be referred to by a plethora of acronyms. Therefore, our study may have merged multiple authors into one person and split journals into multiple entities.

### Practical and Research Implications

#### Practical Implications

This study demonstrates the feasibility of AI-based, largely automatic data mining of large corpora of academic publications. Given the pace and dynamics at which COVID-19 research is being conducted at this time, attempts to manually survey the literature will almost certainly fall behind the state-of-the-art, *unless* specific and confined subtopics are under scrutiny. Automatic data mining, on the other hand, is hindered by the inherent noise in the data (eg, lack of ORCIDs and inability to track genesis and evolution of articles properly). This means that results accurate to every single paper and author are impossible to extract, unless publishers (eg, using blockchain technology) develop a way to trace genesis and evolution of individual publications and unique authors. It is therefore important to stress that such automatic textual analysis is performed *at scale*. If conducted at scale, we would argue that exact numbers may not matter as much as they once may have, considering that being off by as many as 30 individual publications while screening of almost 29,000 publications means that being off by as many as 30 individual publications is would still constitute a negligible fraction of the overall corpus. We, therefore, believe the numbers we have presented in this report to be *aggregates* that are representative of a vibrant and rapidly evolving research landscape and that they highlight trends and shifting interests in topics. Whereas automated data mining excels at providing up-to-date, broad overviews of the field as a whole, manual surveys excel at providing detailed overviews of specific topics. We, therefore, see this study complementing previous manual reviews.

#### Research Implications

This study highlighted the effectiveness of AI methods in the analysis of a large corpus of literature, which researchers can use to perform machine learning–based bibliometric analysis of eHealth-related literature to explore the use of eHealth technologies for COVID-19.

Among the research themes summarized in Table 6, we see the direst need for more research in the “Therapeutics” theme, as clinical aspects and epidemiological aspects are better understood and best practices continue to be more commonly implemented. Consequently, we see this shift in the proportion of articles at the expense of the “Clinical Aspects” and “Epidemiology” themes, as well as the “Prevention” theme. The reason is that the impact of related topics “Social distancing” and “Personal preventive equipment” should be well understood and implemented by now.

In the context of performing bibliometric reviews based on automatically extracted topics, the most important research challenge is to develop methods that are more robust against noise and can process not only abstracts, titles, and author lists, but the entire full text of the publications. Although computationally extremely demanding, this would allow assessing any overlaps between publications, which is a stronger measure than the binary decision of duplicity.

### Conclusions

This study provides a comprehensive overview of the COVID-19 literature. Specifically, we identified the main COVID-19–related topics addressed in the existing literature; weekly trends of publications; and top countries, authors, and publishers. This study will help the research community to understand the evolution of the COVID-19–related literature; prioritize research needs; and recognize the leading researchers, institutes, countries, and publishers for each topic. AI-based bibliometric analysis has the potential to rapidly explore large corpora of academic publications. Publishers should avoid noise in the data by developing a way to trace the evolution of individual publications and unique authors.

## Appendix

Multimedia Appendix 1 Number of publications for each topic.

Multimedia Appendix 2 Number of publications for each theme.

## Abbreviations

AI: artificial intelligence
CORD-19: COVID-19 Open Research Dataset
CSV: comma-separated values
JSON: JavaScript Object Notation
PCR: polymerase chain reaction
TF-IDF: term frequency–inverse document
