# Supplementary material for: A Comprehensive Overview of the COVID-19 Literature: Machine Learning–Based Bibliometric Analysis
Source: J Med Internet Res. 2021 Mar 8;23(3):e23703. doi: 10.2196/23703 (PMC7942394; doi:10.2196/23703)
Supplement: Multimedia Appendix 1 [file jmir_v23i3e23703_app1.docx]

# Appendix 2: Number of publications for each topic

The following figures depict the relative number of publications for each **topic** (blue foreground) over time, compared with the relative number of the publications for all topics (orange background) over time. Trends within each topic compared to the overall research landscape can easily be identified.
