# Supplementary material for: A Comprehensive Overview of the COVID-19 Literature: Machine Learning–Based Bibliometric Analysis
Source: J Med Internet Res. 2021 Mar 8;23(3):e23703. doi: 10.2196/23703 (PMC7942394; doi:10.2196/23703)
Supplement: Multimedia Appendix 2 [file jmir_v23i3e23703_app2.docx]

# **Appendix 3: Number of publications for each theme**

The following figures depict relative number of publications for each **theme** (blue foreground) over time, again compared with the relative number of the publications for all topics (orange background) over time. We note that research interest in Epidemiology and, more recently, Prevention has declined, whereas Clinical Aspects, Therapeutics, and Diagnostics follow the global trend reasonably well. Surprisingly, the theme Related Conditions receives more attention now, after initially lagging behind. It is also interesting that the theme Prevention (like Related Conditions) picked up momentum relatively late, then exceeding the global research trend before settling again. At the beginning of the epidemic, research in the Epidemiology and Diagnostics themes dominated the global trend, which is not surprising as scientists tried to diagnose the new disease and model its spread.
